# Magneto-Elastic μ-Vibrator for Smashing Thrombus

**DOI:** 10.3390/mi10010074

**Published:** 2019-01-21

**Authors:** Wei Li, Cong Xue, Xinxin Li

**Affiliations:** 1 State Key Lab of Transducer Technology, Shanghai Institute of Microsystem and Information Technology, Chinese Academy of Sciences, Shanghai 200050, China; weili@mail.sim.ac.cn (W.L.); xuecong2003@163.com (C.X.); 2 University of Chinese Academy of Sciences, Beijing 100049, China

**Keywords:** micromechanical resonator, biomedical actuator, magneto-elasticity, thrombus clean-up

## Abstract

A miniaturized thrombus dredger is proposed and developed in this study. The flexural resonance of the µ-resonator dredger is driven by a bulk-extensional magneto-elastic vibrator that is externally excited by alternating magnetic-field. With the fabricated prototype of the resonant dredger, a mice thrombus blocked in a simulated vessel is broken into micro-pieces, and the previously blocked vessel can recover to an unobstructed state within 1 h. A flow-rate ratio detection method is used to evaluate the thrombus-cleaning effectiveness. The comparison between the finite-element simulation and the experimental results validates the flow-rate ratio detection method. By optimally exciting the resonant dredger in its third resonant mode, the flow-rate ratio in the cleaned vessel increases by about 2.7 times compared with that in the partly blocked vessel, and the thrombus is smashed into micro-pieces.

## 1. Introduction

Due to contemporary human life habits and increased speed of peoples’ working rhythm, the number of patients who suffer thrombotic diseases has significantly increased in recent years. Thrombotic diseases severely threaten human health, decrease life expectancy, and bring about a heavy burden to the families and to society. At present, the most used medicinal treatment methods, such as tissue plasminogen activator (tPA), still have side effects, and possibly bring about bleeding complications, like neurodegeneration or ischemic stroke [1,2]. Besides, tPA treatment normally takes more than 3 h to dissolve thrombus. The long treatment period is also a potential threat to the patient. Therefore, it is highly important to develop novel technology for rapid breaking thrombus.

Developing a micro-robot to mechanically clean the thrombus in blood-vessels has been a dream for a long time. Researchers and medical workers have also made a great deal of effort, including development of the mechanical embolus removal in cerebral ischemia (MERCI) retriever, intravascular stent, and micro-mechanical mixer. As a medical device for vascular reconstructive surgery, the MERCI retriever is used to remove the blood clots that cause the obstruction with a helical coil formed at the distal end. When the retriever is inserted into the clot site, the helical coil wraps around the clot and allows the clot to be removed [3,4,5,6]. An intravascular stent is a metal or plastic tube inserted into the blocked blood vessel to expand the vessel for promoting blood flow [7,8,9,10]. Unfortunately, the aforementioned methods may cause different degrees of vascular intimal injury, thereby limiting wide clinical applications. Rather than a silicon electrostatic micro-motor, which features too weak loading forces to clean the thrombus, piezoelectric linear-motors were selected for this job [11,12,13,14]. Unfortunately, the developed piezoelectric dredgers generally proved to be incompetent since they suffered in vivo intervention of cable, high driving-voltage, and inadequate force output.

**Figure 1:**
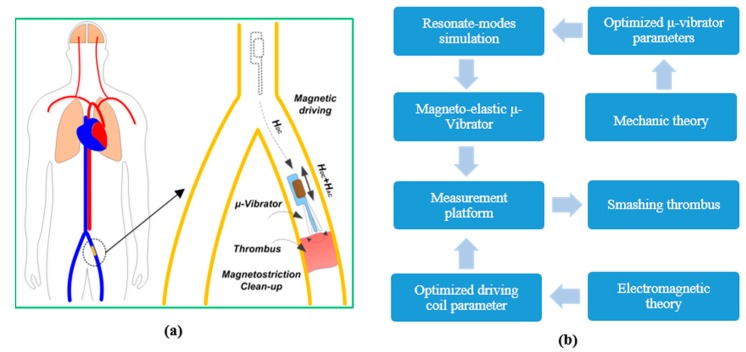
(**a**) Schematic showing thrombus clean-up with the magneto-elastically driven resonant dredger. (**b**) Flowchart showing the design scheme of the thrombus clean-up system.

The functional material of giant magnetostrictive material (GMM) [15] features much greater force output and can be wirelessly driven into mechanical vibration by an electromagnetic field [16,17,18]. The earliest application of GMM was underwater acoustic sonars, which have shown excellent performance. Nowadays, this kind of material has been widely used in the fields of machinery, electronics, petroleum, textile, medical treatment, precision control, and especially ultrasonic applications [19,20,21,22,23,24]. In this study, a miniaturized resonant thrombus dredger is developed, which is driven by GMM bulk-extensional magneto-elastic actuation under an external magnetic field. As is shown in Figure 1, a magneto-elastically driven swing resonator may swim to the thrombus location in the vessel by in vitro magnetic-field driving. Then, with in vitro alternating electromagnetic excitation, the resonating dredger can mechanically shatter the thrombus that obstructs the blood vessel. The design scheme of the thrombus clean-up system is schematically shown in Figure 1b. In following sections, the design of the resonating dredger will be described. Then the experimental results of the fabricated resonating dredger, using a flow-rate ratio measurement method, will be addressed in Section 3. The conclusions will be given in Section 4.

## 2. Design

**Figure 2:**
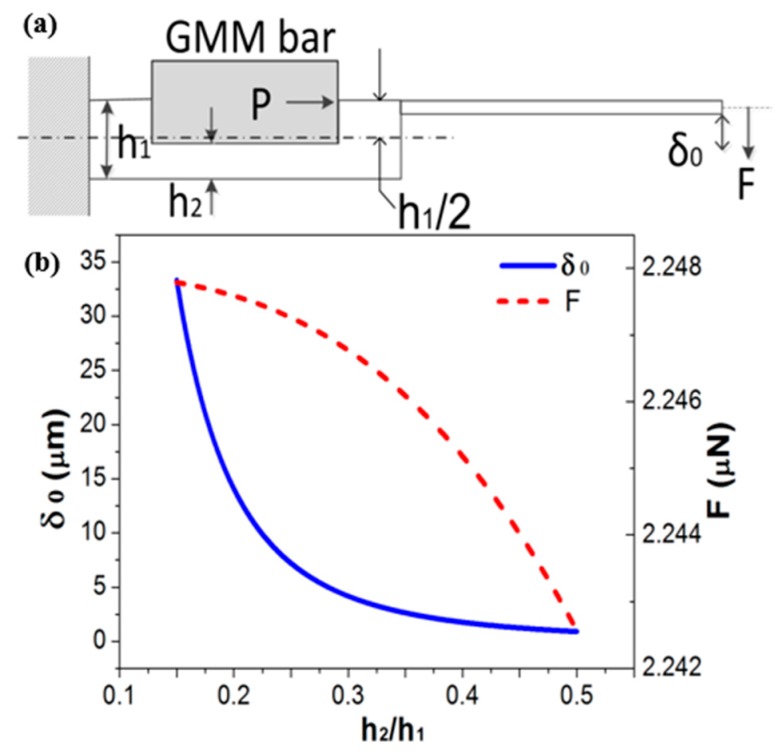
(**a**) Cross-sectional schematic of the resonant dredger structure. (**b**) At the resonator tip, swing-amplitude and output-force versus the groove depth ratio (*h*_2_/*h*_1_) are calculated and plotted, respectively.

Figure 2a schematically shows the design of the resonant dredger, which is composed of three parts: a titanium-made swing cantilever, a base rod with a groove, and a GMM bar. The GMM bar is partly embedded in the groove of the titanium base rod. Purchased from China Iron and Steel Research Institute Group (Beijing, China), the GMM is made of Tb-Dy-Fe alloy that features large magnetostriction (800~1200 × 10^−6^) and high Curie temperature (320~450 °C). The titanium swing cantilever is clamped at the end of the base rod. When the resonant dredger is put in blood vessel, the GMM bar can be in vitro driven into extensional vibration by using an external electromagnetic coil. The longitudinal vibration of the bar generates pulsed force *P*, and thus, the generated pulsed force-moment will bend the groove-contained asymmetric structure. Accordingly, the tip of the cantilever will transversely swing to break the thrombus into pieces. Since the force from extension of the GMM bar is strong enough, the output-force at the displacement-magnified tip is large enough for dredging and smashing thrombus.

For the structure shown in Figure 2a, the maximum GMM-bar driven cantilever-tip displacement (*δ*_0_) can be derived based on mechanics theory, which is

(1) $$\delta_{0}=\frac{M[l_{2}{}^{2}-l_{1}{}^{2}+2\left(l_{2}-l_{1}\right)l_{3}]}{2EI_{2}}$$

where *M* is defined as the GMM-bar vibration induced force-moment, *E* is Young’s modulus of titanium, *l*_1_ is length of the GMM bar, *l*_2_ is length of the base rod, *l*_3_ is length of the vibration bar, and *I*_2_ is the moment of inertia for the base rod. Since the cantilever is operated in resonating vibration, the tip displacement will be further enlarged by the resonating *Q* factor.

Due to the strong force effect from the extensional GMM bar to the end of the base rod, the output force of the dredger can be sufficiently large. Generated at the cantilever tip, the maximum dredging force *F* is achieved when the displacement $\delta_{0}$ = 0. Therefore, the *F* value can be designed according to the equation of

(2) $$F=\frac{3M\left(l_{2}{}^{2}-l_{1}{}^{2}\right)}{2[\left(l_{2}{}^{3}-l_{1}{}^{3}\right)+\frac{h_{2}{}^{3}l_{1}{}^{3}}{h_{1}{}^{3}}]}$$

here *h*_1_ is the thickness of the base rod, *h*_2_ is depth of the groove for accommodating the GMM bar. Both the force-moment *M* = *Ph*_1_/2 and the moment of inertia of the base rod *I*_2_ = *wh*_2_^3^/12 (*w* is width of the base rod) are closely related to the groove depth-ratio of *h*_2_/*h*_1_. For optimal design of the resonant dredger, the vibration-amplitude (*δ*_0_) and the force (*F*) versus the *h*_2_/*h*_1_ are calculated and shown in Figure 2b, respectively. For optimal design, we hope the force can be large enough, but the amplitude should be moderate to avoid excessive displacement induced damage to the vessel wall. Hence, the comprehensively optimized *h*_2_/*h*_1_ ≈ 0.15 is chosen, where *F* is nearly saturated and the displacement is appropriate.

**Figure 3:**
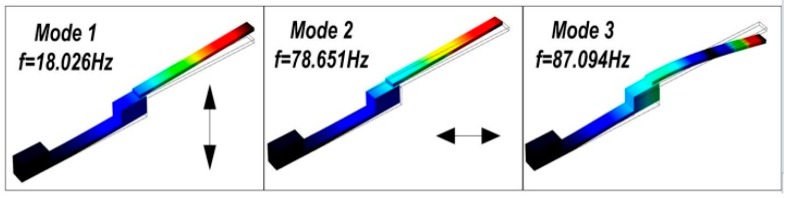
Finite-element analysis results for the earliest three resonance modes of the flexural cantilever. The first and the third modes are for vertical vibration, but the second one is for horizontal movement.

Simulated by using the finite-element software of COMSOL (COMSOL AB, Stockholm, Sweden), the earliest three resonance-modes and the corresponding frequencies of the cantilever dredger are simulated, with the mode-shapes shown in Figure 3. The first mode and the third mode resonances are both along vertical direction, with the resonant frequencies as 18.026 Hz and 87.094 Hz, respectively. In contrast, the second mode resonance at 78.651 Hz has horizontal movement. Driven at different mode frequencies, the thrombus cleaning performance of the resonant dredger will be experimentally compared in the next section.

## 3. Experimental Results

**Figure 4:**
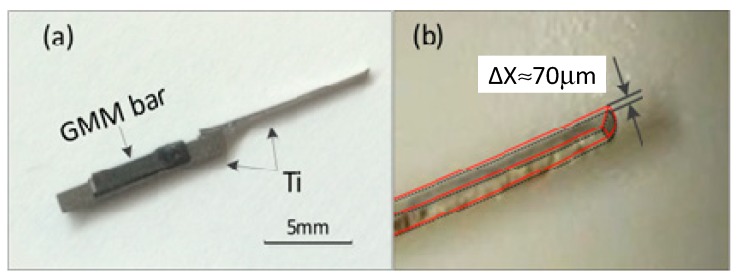
(**a**) Photograph of the fabricated thrombus dredger. (**b**) Cantilever tip vibration formed blur is recorded by the video taken under an endoscope (purchased from Shanghai AOHUA Photo-electricity Endoscope Co. Ltd.). The endoscope is used to locally monitor the vibration.

The proto-typing thrombus dredgers are fabricated according to the optimal design. The 10 mm × 1 mm × 1 mm outer-contoured base rod is made of titanium-alloy. The groove is formed by electrical discharge machining (EDM), with the fabrication precision tolerance being less than 1 µm. The 10 mm × 1 mm × 0.2 mm size vibratory cantilever is also made of Ti-alloy, and welded at the end of the base rod. The GMM bar, of 5 mm × 1 mm × 1 mm in dimension, is tightly embedded in the groove of the base rod. The photo-picture of the fabricated thrombus cleaner is shown in Figure 4a. In the vibration experiment, the vibratory device is put into the air core of an electro-magnetic coil, so as to be driven by a 200 Oe AC magnetic field that is parallel to the GMM bar. A commercial endoscope (made by Shanghai AOHUA Photo-electricity Endoscope Company, Shanghai, China) is inserted into the coil to record the vibration of the titanium-alloy cantilever. Figure 4b shows the endoscope video recorded blur of the third mode resonance at the cantilever tip. The 87 Hz displacement blur ∆*x* at the cantilever tip is measured as about 70 μm. Thus, the resonance *Q* factor should be about 2.

**Figure 5:**
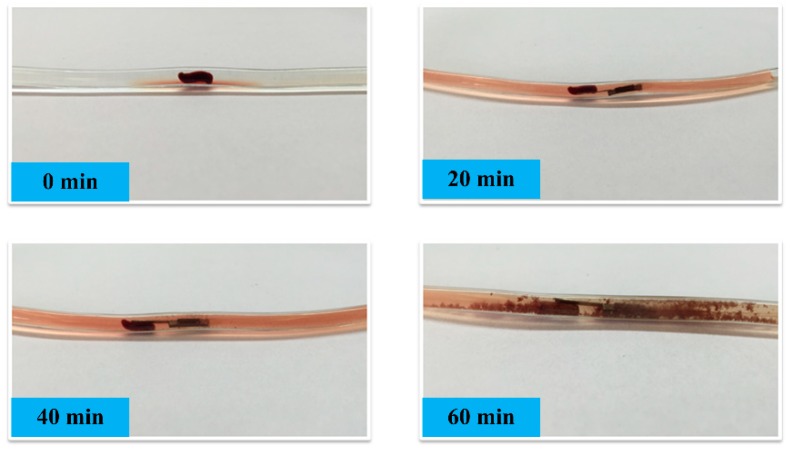
Photos to record the mice thrombus cleaning process, by using the dredger resonance for 0 min, 20 min, 40 min, and 60 min, respectively.

To visually evaluate the thrombus cleaning performance of the device, a piece of mice thrombus is intentionally blocked in a simulated blood vessel that is filled with physiological saline. The mice thrombus is provided by the Medical College of Fudan University, Shanghai. The simulated vessel is made of silicone, with an inner diameter as 3 mm. After the thrombus dredger is put inside the vessel, it is driven to approach the thrombus and begins to vibrate under electro-magnetic excitation of the external coil. The digital images in Figure 5 recorded the mice thrombus cleaning process, where the image recording interval is 20 min. Herein, the vibration is driven under 87 Hz, which is the third mode resonant frequency of the cantilever. Along with the resonating period of time, the mice thrombus is gradually smashed into tiny pieces and the vessel recovers to an unobstructed state within 1 h.

**Figure 6:**
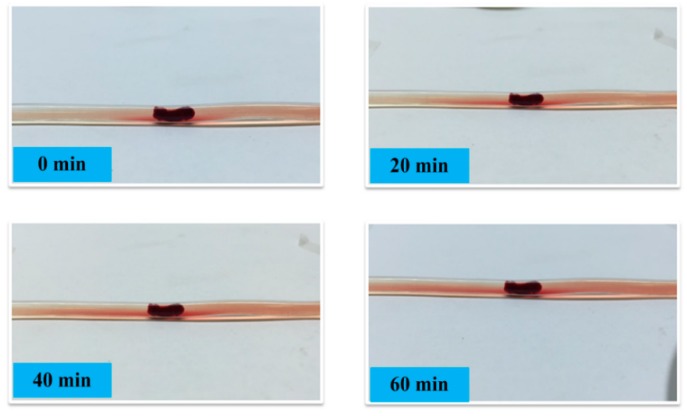
Photos to record the control experiment process. The thrombus statically stays in the vessel for 0 min, 20 min, 40 min, and 60 min.

A control experiment is also carried out. During the same period of 1 h, the thrombus is statically immersed in the physiological-saline filled vessel (without the dredging). The situations at identical moments are recorded in Figure 6 for comparison. Without dredger vibration, the mice thrombus has no significant change in both shape and size.

**Figure 7:**
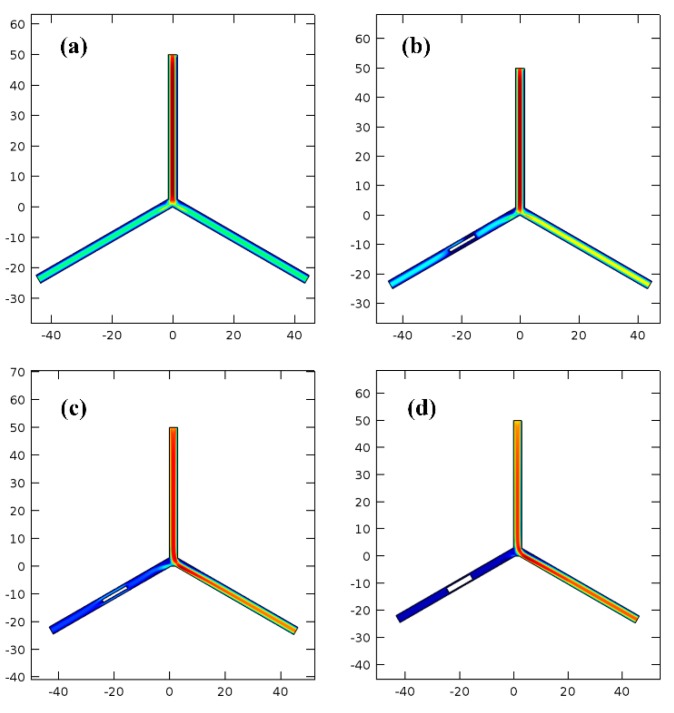
Finite-element simulation results for the flow-rate ratio. (**a**) No plug in the left branch, (**b**–**d**) 1 mm, 1.5 mm, and 2 mm diameter plug in the left branch. All the length data in *X* and *Y* axes are in millimeter.

In order to evaluate the thrombus cleaning effectiveness, a flow-rate ratio measurement method is proposed. As is illustrated in Figure 7, water flow-rate in a three-way channel is simulated with the finite-element analysis software of COMSOL. The different colors represent different flow rates. The color change from blue to red indicates increase of the flow rate. If the two branches are both unobstructed, the flow rates in the two branches are the same (see Figure 7a). However, when the left branch of the three-way channel is partly blocked by inserting a solid plug, the flow rates in the two branches will be different. Herein, the flow rate in the blocked branch divided by the flow rate in the other branch is defined as flow-rate ratio. As is shown in Figure 7b–d, the plugs with various diameters are sequentially inserted into the left branch to simulate different degrees of blood-vessel embolism. The flow-rate ratios are simulated accordingly.

**Figure 8:**
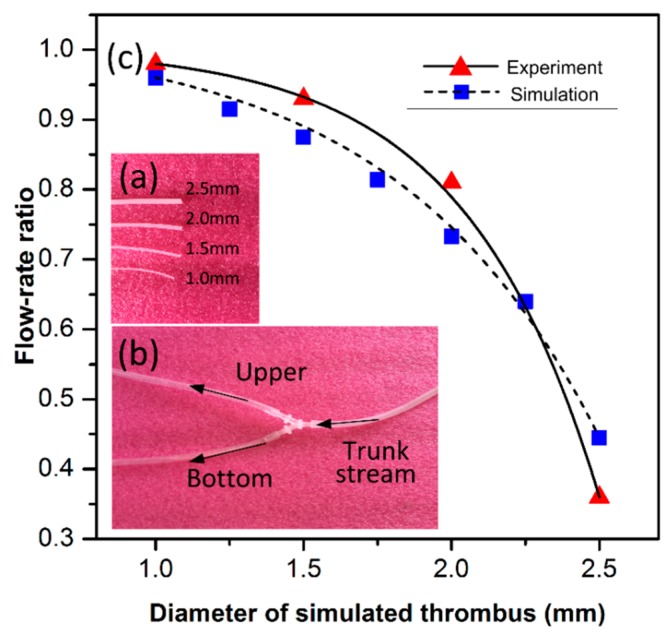
(**a**) Four solid silicone bars with different diameters. (**b**) Three-way simulated vessel used for experimental evaluation of thrombus cleaning performance by measuring the flow-rate ratio between the two branches, where the upper branch is partly blocked by sequentially inserting the bars. (**c**) The results from the experiment and the simulation generally agree, and both indicate that the flow-rate ratio increases along with decreasing the plug diameter.

The flow-rate ratio is also experimentally obtained. In the upper branch of the three-way (see Figure 8c), four solid silicone bars are sequentially plugged in. The four bars have identical lengths of 1 cm but different diameters of 2.5, 2.0, 1.5, and 1.0 mm (see Figure 8b) to simulate different degrees of blockage against the physiological-saline flow in the 3 mm inner-diameter vessel. The bottom branch of the three-way is always kept unblocked. The flow-rate ratio between the upper and the bottom branches, named *r*_upper-branch_/*r*_bottom-branch_, is measured to evaluate the thrombus cleaning effectiveness. During the measure experiment, the flow-rate of the trunk stream is always retained as a constant. Along with the diameter of the inserted silicone bar successively decreasing from 2.5 mm to 1 mm, the measured *r*_upper-branch_/*r*_bottom-branch_ increases from 0.36 to 0.98. The results from the experiment and the simulation are shown in Figure 8c, where the slight difference between them is mainly caused by the measurement setup conditions, which are not ideal. The results validate the assessment method by measuring that the flow-rate ratio can be feasibly used to experimentally evaluate thrombus cleanup performance.

**Figure 9:**
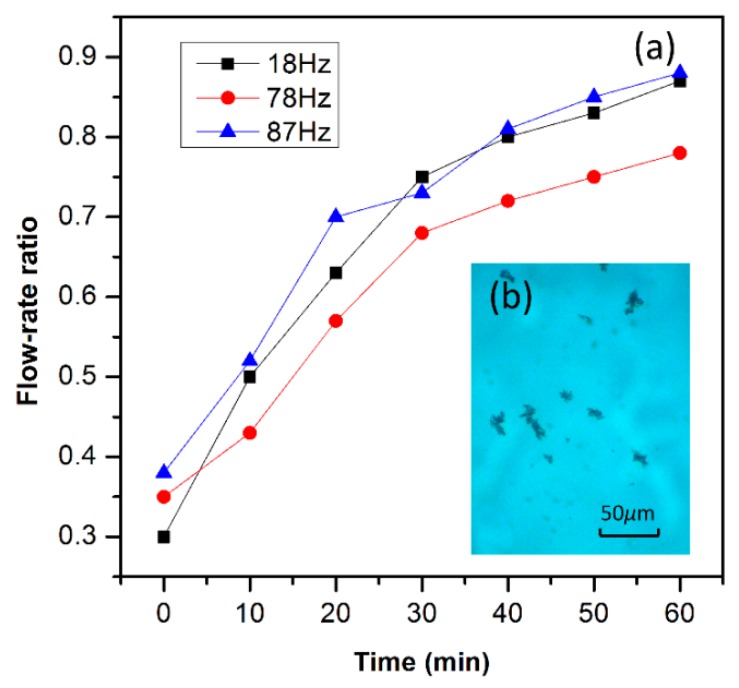
(**a**) Comparison among the tested flow-rate ratio values for the vibrator working in the earliest three resonance modes. (**b**) Micrograph showing that the thrombus has been smashed into tiny pieces of less than 30 μm.

By using the flow-rate ratio assessment method, we experimentally compared the thrombus cleaning efficiency under different resonant modes of the vibratory dredger. By sequentially driving the device to resonate in its earliest three resonance-modes, respectively, the mice thrombus cleaning experiment is implemented in the three-way simulated vessel. Similar sized thrombus pieces are taken from the same thrombus sample to examine the cleanup performance by using different resonance modes. The measured flow-rate ratios at various dredging moments are all plotted in Figure 9a. Obviously, operation at the second mode frequency exhibits the worst thrombus cleaning performance. In contrast, the resonant dredger working at the frequency of the first and the third modes exhibit better efficiency. It can be explained that the lateral displacement of the cantilever in the second mode is too small to effectively smash the thrombus. In contrast, the larger vertical vibration amplitude in either the first or the third mode facilitates cleaning of the thrombus. After the vibrator resonates in the third mode for 1 h, the experimental results demonstrate that the flow-rate ratio increases from 0.38 up to 0.87, due to the cleanup of the massive thrombus. After the cleanup for 1 h, the micrograph of Figure 9b shows that the mice thrombus has been shattered into tiny pieces, with the size generally less than 30 μm.

## 4. Conclusions

A novel magnetelastic-driven miniature thrombus dredger is proposed, designed, and fabricated for breaking thrombus in blood vessel. The structure is optimally designed to achieve efficient thrombus cleanup performance. The mice thrombus blocked in the simulated vessel is experimentally broken into micro-pieces, and the vessel can recover to an unobstructed state within 1 h. In order to evaluate the device, we define the flow-rate ratio between the two branches of a three-way simulation vessel, where one branch can be partly blocked at various degrees. By exciting the vibrator in its third resonating mode, the highest dredging effectiveness is achieved. The flow-rate ratio in the cleaned vessel increases by about 2.7 times compared with that in the partly blocked state, and the massive thrombus is shattered into tiny pieces, with the size range within tens of microns. Such tiny pieces are easily drawn out of the vessel by interventional medical treatment. Compared with the reported thrombus dredgers [11,12,13,14], the device in this work exhibits comprehensive superiority in miniaturization, dredging effectiveness, and wireless driving. Therefore, the novel thrombus dredger in this paper is promising to be further developed into clinical application of rapid thrombus clearance.
